# A PIP5 Kinase Essential for Efficient Chemotactic Signaling

**DOI:** 10.1016/j.cub.2013.12.052

**Published:** 2014-02-17

**Authors:** Louise Fets, John M.E. Nichols, Robert R. Kay

**Affiliations:** 1 Cell Biology Division, MRC Laboratory of Molecular Biology, Cambridge Biomedical Campus, Francis Crick Avenue, Cambridge CB2 0QH, UK

## Abstract

In neutrophils and *Dictyostelium*, chemoattractant gradients generate directed cell migration by eliciting signaling events that bias intrinsic motility and favor the production and retention of upgradient pseudopods [1, 2]. Phosphoinositides are actively regulated during chemotaxis in these cells, most iconically in the production of PI(3,4,5)P_3_ gradients within the plasma membrane [3, 4]. Although it is now known that PI(3,4,5)P_3_ signaling is nonessential for gradient sensing [5, 6], the role of the related phosphoinositide PI(4,5)P_2_ is little understood, despite its clear importance in many cell biological processes [7]. We describe here a PIP5 kinase, PikI, which produces PI(4,5)P_2_ and is essential for efficient chemotaxis of *Dictyostelium* cells. Without PikI, PI(4,5)P_2_ levels are reduced by 90%, and while *pikI*^−^ cells move at normal speeds, they are highly disorientated in cAMP gradients. Following chemotactic stimulation, Ras is efficiently activated in *pikI*^−^ cells, yet Ras-dependent responses (including activation of PKB) are severely impaired. PikI is phosphorylated by PKB [8], and in vitro studies of a phosphomimic mutant suggest that this phosphorylation increases PikI activity. We propose that adequate PI(4,5)P_2_ levels are required to couple activated Ras to its downstream effectors and that these levels are regulated by PikI, making it a crucial player in gradient sensing.

## Highlights

• Gradient sensing is severely impaired in *pikI*^−^ cells, but cell speed is unaffected
• PikI is the major PIP5K in *Dictyostelium* and produces 90% of cellular PI(4,5)P_2_
• PikI is required for active Ras to couple to downstream effectors such as TorC2
• Phosphorylation by PKB (downstream of Ras) may increase PikI activity

## Graphical Abstract

**Figure:**
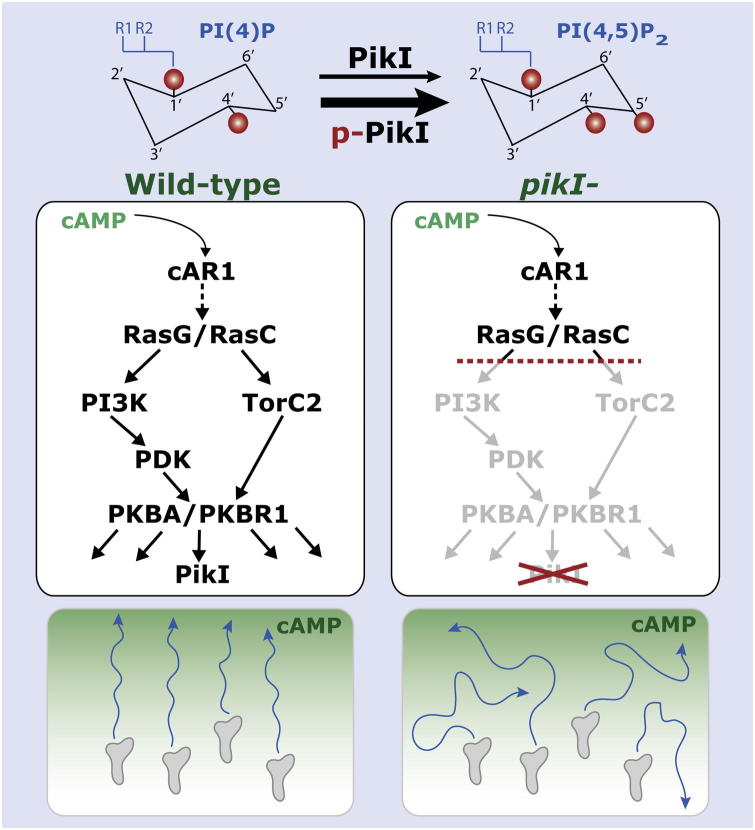

## Teaser

Fets et al. show that PikI, a PIP5 kinase that generates PI(4,5)P_2_, is vital for chemotaxis in *Dictyostelium* cells. Without PikI, cells are able to move at normal speeds, but gradient sensing is dramatically attenuated as a result of uncoupling of activated Ras from its downstream effectors.

## Results and Discussion

### PikI Is Required for Directional Accuracy, but Not General Motility

The identification of a PIP5 kinase (PIP5K) that is strongly phosphorylated by PKBA and PKBR1 within seconds of stimulation with the chemoattractant cAMP [8] suggested that regulation of PI(4,5)P_2_ levels may play a role during chemotaxis in *Dictyostelium*. To investigate this, we created a strain in which this gene is deleted from the wild-type strain, Ax2 (see Figures S1A–S1C available online). The protein was named PikI (phosphatidylinositol kinase I; gene *pikI*, DDB_G0267588). The *pikI*^−^ strain has a severe growth defect in liquid medium and impaired phagocytosis (Figures S1D–S1F). Development is also affected, and *pikI*^−^ cells fail to aggregate on bacterial (Figure S1E) and nonnutrient agar plates (data not shown).

**Figure 1:**
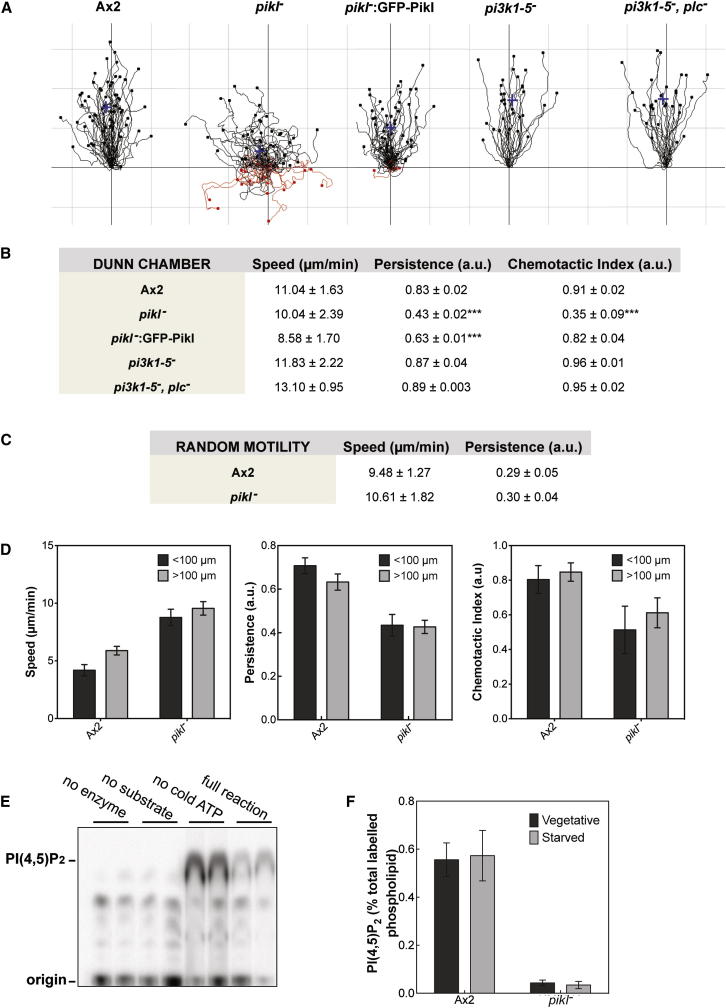
Loss of the Active PIP5K, PikI, Severely Impairs Gradient Sensing (A) Representative tracks of cells (strains indicated) chemotaxing toward cAMP in a Dunn chemotaxis chamber. Red tracks represent cells that have moved downgradient overall. Blue crosses represent the center of mass of the tracks. Grid lines are 100 μm apart. (B) Table of chemotactic parameters obtained by tracking cells in a Dunn chemotaxis chamber. Speed is defined as accumulated distance divided by time, persistence as the Euclidean distance divided by accumulated distance, and chemotactic index as the cosine of the angle between the net distance traveled in the direction of the gradient and the Euclidean distance. Data represent the grand mean ± SD and were calculated from mean values obtained on at least two different days (Ax2: 6 days, n = 350 cells; *pikI*^−^: 3 days, n = 173 cells; *pikI*^−^: GFP-PikI: 3 days, n = 173 cells; *pi3k1*^−^*5*^−^: 4 days, n = 120; *pi3k1*^−^*5*^−^*,plc*^−^: 2 days, n = 60 cells. Means for each day were compared using a one-way ANOVA with Tukey’s post hoc test. ^∗∗∗^p < 0.001, mutant and rescued strains versus wild-type. (C) Table of vegetative random motility parameters. Data represent the grand mean ± SD and were calculated from mean values obtained on three different days (Ax2 and *pikI*^−^, n = 60). (D) Graphs representing speed, persistence, and chemotactic index of cells moving toward a micropipette filled with 10 μM cAMP (strong gradient). The chemotactic abilities of cells closer to the micropipette (<100 μm) were compared with those further from it (>100 μm). Data represent the mean ± SEM from three different days (Ax2 < 100 μm, n = 14; Ax2 > 100 μm, n = 45; *pikI*^−^ < 100 μm, n = 17; *pikI*^−^ > 100 μm, n = 42). (E) Thin-layer chromatography separation of products from a PIP kinase assay, showing that recombinant PikI is an active PIP5K. (F) Quantification of PI(4,5)P_2_ levels in cells labeled with ^32^P_i_ over several generations to reach steady-state labeling. PI(4,5)P_2_ levels in both vegetative and 5 hr-starved cells were compared. Data represent mean ± SEM calculated from at least three independent experiments. See also Figure S1 and Movie S1.

Chemotaxis of *pikI*^−^ cells toward cAMP was analyzed using a Dunn chamber [9]. Mutant cells have dramatic defects in chemotactic index and directional persistence (Figures 1A and 1B), which can be corrected by overexpression of GFP-PikI. Strikingly, despite this reduced chemotactic index, the speed of *pikI*^−^ cells is indistinguishable from wild-type (Figures 1A and 1B; Movie S1), demonstrating that although PikI is necessary for efficient gradient sensing, it is dispensable for general motility. Indeed, randomly moving vegetative *pikI*^−^ cells exhibit wild-type speed and persistence (Figure 1C). Chemotaxis in steeper gradients was examined using a cAMP-filled micropipette (Figure 1D). Although the chemotactic index of *pikI*^−^ cells was slightly improved over that in a shallow gradient, there was no effect of initial distance from the cAMP source. Interestingly, the speed of *pikI*^−^ cells was faster than that of Ax2 when chemotaxing toward the micropipette.

PikI is predicted to produce PI(4,5)P_2_, the substrate for PI3 kinases (PI3Ks) and phospholipase C (PLC), both of which are active in chemotactic signaling. To address whether the *pikI*^−^ phenotype results from reduced signaling via these enzymes, we knocked out *plcA* in the *pi3k1*^−^*5*^−^ strain [5]. Chemotaxis in both the *pi3k1*^−^*5*^−^ and the *pi3k1*^−^*5*^−^*,plcA*^−^ strains was found to be as efficient as wild-type (Figures 1A and 1B). Similarly, loss of PLA_2_ [10] in the *pi3k1*^−^*5*^−^ strain caused no further chemotactic defects (O. Hoeller, personal communication). This suggests that disruption of these pathways cannot explain the severity of the *pikI*^−^ phenotype and that PI(4,5)P_2_ is playing another, more important role in gradient sensing.

*Dictyostelium* become competent to chemotax toward cAMP after starvation, which leads to expression of developmentally regulated genes such as the cAMP receptor (*carA*, a G protein-coupled receptor, protein name cAR1). We found that expression of the developmentally regulated gene contact sites A (*csaA*) was normal in *pikI*^−^ cells (Figure S1H), while levels of the cAMP receptor are at least as high in *pikI*^−^ as in wild-type cells (Figure S1G). The chemotactic defects of *pikI*^−^ cells are therefore likely due to a defect in chemotactic signal transduction rather than a general developmental defect. Finally, among the cAMP responses tested (see below), we found that phosphorylation of Erk2 is normal in *pikI*^−^ cells (Figure S1I), confirming that the cAMP receptor is active. Unusually, Erk2 phosphorylation requires the cAMP receptor but is partially independent of the coupled heterotrimeric G protein [11].

### PikI Is an Active Type 1 PIP Kinase

The activity of purified recombinant PikI was measured using a range of substrates (Figures 1E and S1J). PikI preferentially phosphorylated PI(4)P, showing that it is an active type 1 PIP5K. PikI could also phosphorylate PI(3)P and, unusually, PI(5)P, demonstrating weak type 2 activity.

PikI is one of seven PIP kinases in *Dictyostelium.* We used in vivo labeling with ^32^P_i_ to assess the contribution of PikI to overall PI(4,5)P_2_ production. During short-term labeling, the rate of PI(4,5)P_2_ labeling was dramatically reduced in *pikI*^−^ cells, whereas bulk phospholipid labeling was unaffected (Figures S1J and S1K). Longer-term labeling revealed that steady-state levels of PI(4,5)P_2_ in *pikI*^−^ cells are 10-fold lower than in wild-type cells (Figure 1F), indicating that PikI is responsible for the bulk of PI(4,5)P_2_ synthesis. This supports RNA sequencing expression data showing that PikI is highly expressed throughout development and is the most highly expressed PIP5K during vegetative growth (data from http://dictyexpress.biolab.si/). None of the other labeled phospholipids were significantly altered (Figures S1M and S1N). These results demonstrate that PikI, and therefore PI(4,5)P_2_ itself, plays a crucial role in chemotactic gradient sensing.

### Loss of PikI Causes a Dramatic Reduction in Almost All cAMP Responses

**Figure 2:**
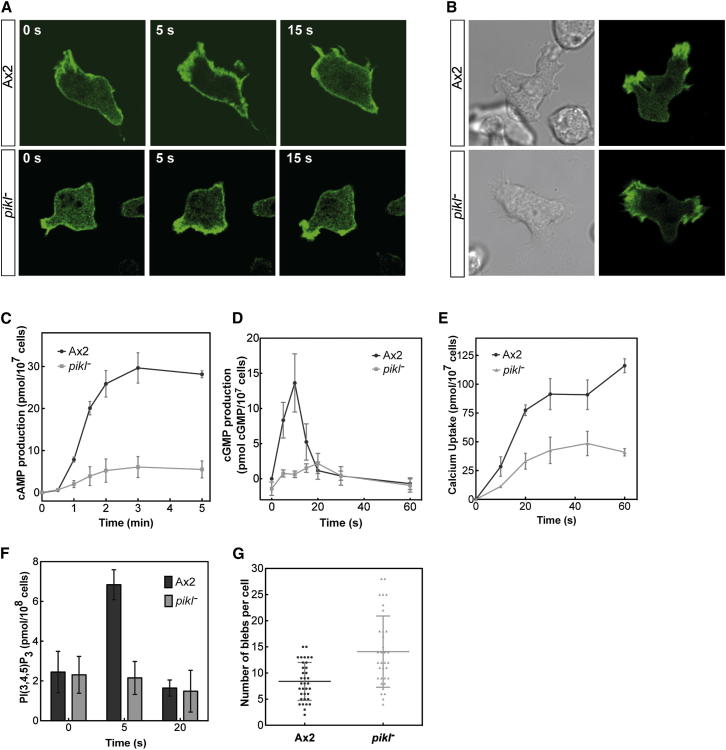
Key Responses to cAMP Are Attenuated in *pikI*^−^ Cells (A) Representative response of Lifeact-GFP-expressing cells to uniform stimulation with cAMP. (B) Lifeact-GFP marking F-actin in pseudopods of randomly moving, chemotactically competent cells. See also Movie S2. (C) cAMP production in response to stimulation with 2-deoxy-cAMP. (D) Production of cGMP in response to cAMP stimulation of cells. (E) ^45^Ca^2+^ uptake by cells in response to cAMP stimulation. (F) PI(3,4,5)P_3_ levels before and after stimulation with cAMP. (G) Quantification of the number of blebs produced per cell in response to cAMP stimulation. All graphs show the mean of at least three experiments conducted on separate days; error bars represent ± SEM.

The PikI null mutant has a severe chemotactic defect, yet it migrates at wild-type speeds, indicating that this defect is due to a specific lesion in signal processing. In order to identify the lesion, we investigated well-characterized responses made by *Dictyostelium* to uniform stimulation with a saturating dose of cAMP. cAMP stimulation of *Dictyostelium* causes a rapid, transient peak of actin polymerization followed by a second, more variable peak and finally a return to basal levels of F-actin. Although this pattern is retained in *pikI*^−^ cells, the magnitude of F-actin accumulation is diminished (Figure S2A). Similarly, the redistribution of Lifeact-GFP upon uniform stimulation of *pikI*^−^ with cAMP (Figure 2A) was also reduced compared with wild-type. Resting F-actin levels also appear diminished (Figure S2B), although noise in the data precludes a significant result. *pikI*^−^ cells are still capable of polymerizing actin, however, as F-actin accumulation can be seen in pseudopods of randomly moving cells (Figure 2B; Movie S2).

During the phase of rapid F-actin depolymerization poststimulation, wild-type cells begin to bleb [12]. The number of blebs observed per cell was higher in the *pikI* mutants than in wild-type (Figure 2G); however, the number of responding cells was reduced. PI(4,5)P_2_ is important for membrane-cortex adhesion [13]; therefore, low PI(4,5)P_2_ levels in *pikI*^−^ cells could reduce the threshold for blebbing, perhaps explaining this increase. cAMP stimulation also elicits second-messenger responses in wild-type cells [14]. We found that both cAMP and cGMP responses are greatly attenuated in mutant cells (Figures 2C and 2D). Calcium uptake was also reduced in comparison with wild-type (Figure 2E), but to a lesser extent. This response, like ERK2 phosphorylation, is receptor dependent but at least partly independent of heterotrimeric G proteins [15].

PI(3,4,5)P_3_ is produced transiently in the plasma membrane after cAMP stimulation due to PI3K activity [16, 17]. cAMP-induced PI(3,4,5)P_3_ production is absent in the *pikI*^−^ strain; however, basal levels of this lipid appear unaltered (Figure 2F). Attenuation of cAMP, cGMP, calcium, and PI(3,4,5)P_3_ second-messenger responses in the *pikI*^−^ mutant points to a defect high in the signal transduction pathway. Two *Dictyostelium* Akt homologs, PKBA and PKBR1, are central to chemotactic signal transduction, and their loss causes severe chemotactic defects [8]. We therefore examined their activity in the *pikI*^−^ mutant.

### PikI Is Required for Activation of the Akt Homologs PKBA and PKBR1

**Figure 3:**
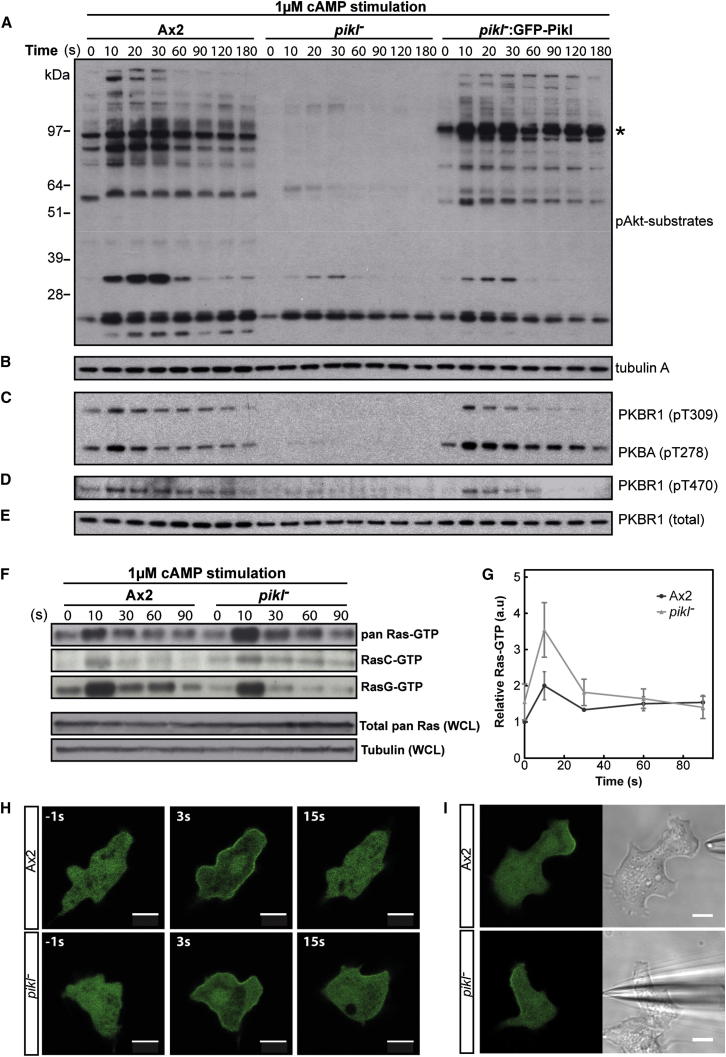
Ras, but Not PKB/PKBR1, Is Efficiently Activated in Response to cAMP in *pikI*^−^ Cells (A–E) Representative western blots showing cell lysates from a time course after cAMP stimulation (at time zero). The primary antibodies listed below were used to blot. (A) Anti-phospho-Akt-substrates (recognizing the phosphorylated substrates of PKBA and PKBR1). The asterisk shows the position of phosphorylated GFP-PikI; the band below 28 kDa is also seen in the *pkbR1*^−^ strain [8] and is likely to be nonspecific. (B) Anti-tubulin A (loading control). (C) Anti-phospho-PKC-pan-zeta (recognizes the phosphorylated activation loop of PKBA and PKBR1). (D) Anti-phospho-PDK1-docking motif (recognizes the phosphorylated hydrophobic motif of PKBR1). (E) Anti-PKBR1 (total PKBR1 levels). (F) Representative western blots showing Ras-GTP pulled down from lysates (400 μg protein per strain) of cells stimulated with cAMP over the time course indicated (n = 3). Blots were probed with anti-pan-Ras, anti-RasC, or anti-RasG as indicated. Two blots also show total Ras and tubulin in whole-cell lysate (WCL) as loading controls. (G) Quantification of Ras-GTP pull-down; quantification shows mean (pan) Ras-GTP levels from anti-pan-Ras blots obtained in three independent experiments after normalization to Ax2 basal levels of Ras. Error bars represent ± SD (n = 3). (H) Representative response of Raf1-RBD-GFP-expressing cells to uniform stimulation with cAMP. (I) Example of localization of Raf1-RBD-GFP in cells responding to a steep cAMP gradient produced by a micropipette. Scale bars represent 5 μm. See also Figure S2 and Movie S3.

PKBA and PKBR1 activity was assayed using an antibody that recognizes their shared phosphorylated substrate motif. In wild-type cells, following cAMP stimulation, multiple proteins are phosphorylated and dephosphorylated with different temporal signatures. In the *pikI*^−^ strain, cAMP-induced substrate phosphorylation is almost entirely lost, with basal substrate phosphorylation also being very low (Figures 3A and S2A). Full substrate phosphorylation is rescued by GFP-PikI expression, and the appearance of a new band corresponding to this fusion protein confirms previous results, showing that it too is phosphorylated [8]. Normal PKBR1 expression in the mutant suggests that the defect lies in activation, not expression, of these Akt homologs (Figure 3E).

Activation of PKBA/PKBR1 occurs by phosphorylation at two sites: the hydrophobic motif (HM), mediated by the TorC2 complex [8], and the activation loop, mediated by the phosphoinositide-dependent kinases PDKA and PDKB [18]. Phosphospecific antibodies showed that phosphorylation of these motifs following cAMP stimulation is dramatically reduced in *pikI*^−^ cells (Figures 3C and 3D). It therefore appears that activation of PDKA and PDKB (via PI3 kinases) and TorC2 is significantly diminished in *pikI*^−^ cells, accounting for absence of phosphorylation and activation of PKBA and PKBR1. Since TorC2 and PI3Ks are activated by Ras [19–21], we next investigated Ras activation in PikI nulls.

### PikI Is Necessary for Signal Transduction through Activated Ras

The two main Ras GTPases involved in chemotaxis are RasC and RasG [22, 23]. RasC lies upstream of TorC2, while RasG is involved in PI3K activation. The Ras-binding domain of Byr2 was used to pull down activated Ras (Ras-GTP) from cAMP-stimulated cell lysates. Surprisingly, not only is Ras activated in the absence of PikI, pan-Ras-GTP levels are also higher at their peak than in wild-type (Figures 3F and 3G). Examination of RasG- and RasC-specific activation showed that peak levels of activated RasG appear unaffected in the mutant, despite reduced levels of RasG-GTP at all other time points (Figure 3F). Levels of RasC activation are slightly increased in the mutant, and basal levels of RasC-GTP are also higher than in wild-type cells.

These results show that Ras is activated in response to cAMP but that the activation of Ras effectors such as TorC2 and PI3K is greatly attenuated. These findings suggest a generic problem in coupling of activated Ras to at least a large subset of its downstream effectors in mutant cells. Since PikI is an active PIP5 kinase and PI(4,5)P_2_ levels are low in PikI null cells, this implies that Ras coupling to its downstream effectors is dependent on PI(4,5)P_2_. Small G proteins containing polybasic clusters of amino acids bind specifically to the plasma membrane using both lipid anchors and electrostatic interactions with anionic lipids [24–26]. Low PI(4,5)P_2_ levels might weaken electrostatic interactions, resulting in mislocalization of Ras and, consequently, uncoupling of activated Ras from its effectors. Cell fractionation was used to examine levels of membrane-bound Ras in resting cells and showed that whereas cytoplasmic levels of Ras are increased in *pikI*^−^ cells, levels of membrane-bound Ras are comparable with wild-type (Figure S3B).

Expression of the GFP-tagged Ras-binding domain of Raf1 [21] showed that localization of active Ras was normal in *pikI*^−^ cells, both after uniform stimulation with cAMP and when exposed to steep cAMP gradients (Figures 3H and 3I; Movie S3). It should be noted that an arc of Ras activation in steep gradients was observed in only some cells in both Ax2 and *pikI*^−^ mutants.

These results are consistent with recent evidence that many proteins that bind to PI(4,5)P_2_ through an electrostatic interaction also interact redundantly with PI(4)P in the membrane [27]. It therefore appears that PI(4,5)P_2_ may play a more specific role in regulating Ras activity, independent of any role in localizing Ras to the plasma membrane, perhaps by directly affecting the ability of Ras to modulate the activity of its effectors through conformational changes.

### Phosphorylation of PikI May Form a Positive Feedback Loop

**Figure 4:**
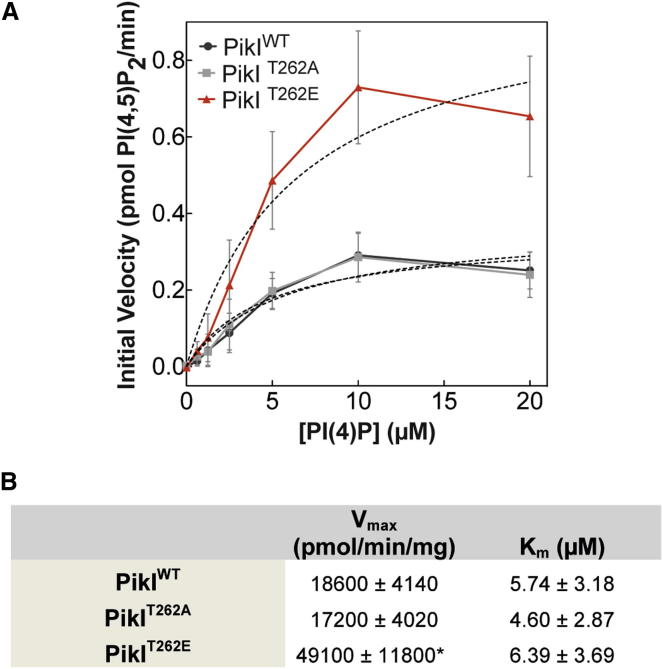
Phosphorylation of PikI Activates Kinase Activity (A) Quantification of PI(4,5)P_2_ production over a range of substrate concentrations for wild-type and phosphomutants of PikI. Data represent mean ± SEM from four repeats using two different recombinant protein preparations. (B) K_m_ (μM) and V_max_ (pmol PI(4,5)P_2_/min/mg recombinant protein) of wild-type and phosphomutants of PikI, determined by fitting Michaelis-Menten curves to each data set and compared using a one-way ANOVA followed by Tukey’s post hoc test. ^∗^p < 0.05.

Our results suggest that PikI is necessary for signaling via PKBA and PKBR1, yet PikI was originally identified as a substrate of these kinases, raising the possibility of a feedback loop. To determine the function of this cAMP-dependent phosphorylation at threonine 262, we generated nonphosphorylatable (PikI^T262A^) and phosphomimetic (PikI^T262E^) point mutants and examined their enzymatic properties. It is apparent that whereas PikI^T262A^ is indistinguishable from PikI^WT^, PikI^T262E^ is significantly more active, with an almost 3-fold increase in V_max_ (Figures 4A and 4B). This suggests that PikI activity is increased as a result of phosphorylation by PKBA/PKBR1 and that this modification of PikI may form part of a positive feedback loop.

We have shown that extreme depletion of PI(4,5)P_2_ severely attenuates Ras signaling, and it is possible that lesser depletions would also be deleterious. Although we cannot detect changes in global PI(4,5)P_2_ levels during cAMP signaling by in vivo labeling with ^32^P_i_ (unpublished data), localized changes in PI(4,5)P_2_ levels could occur, as has been suggested in other systems [28]. PI(3,4,5)P_3_ production (and hence PI(4,5)P_2_ consumption) can be sharply localized to the leading edge during chemotaxis, and PLC activity may be similarly localized [29]. Thus, the leading edge is likely to be depleted of PI(4,5)P_2_. Conversely, PKBR1 is selectively activated at the leading edge [8], and therefore PikI should also be locally phosphorylated and activated. We therefore propose that PikI is activated at the leading edge, thereby sustaining PI(4,5)P_2_ levels in regions of high PI3K and PLC activity and providing a feedback loop governing the strength of chemotactic signaling.

Although PI(4,5)P_2_ regulation during chemotaxis has been studied previously [30–33], our work represents the first evidence that a PIP5K, and therefore PI(4,5)P_2_, plays a vital role in gradient sensing at the leading edge, via manipulation of Ras signaling. This demonstrates for the first time that while PI(3,4,5)P_3_ signaling may be dispensable for chemotactic gradient sensing, maintenance of PI(4,5)P_2_ is vital.

## Supplemental Information

Document S1. Figures S1–S3 and Supplemental Experimental Procedures

Movie S1. Chemotaxis in Wild-Type and *pikI*^−^ Cells, Related to Figure 1 Ax2 and *pikI*^−^ cells chemotaxing toward cAMP in a Dunn chamber (the outer well, filled with 1 μm cAMP, can just be seen at the top of the screen). Acquisition frame rate is two frames per minute.

Movie S2. Actin Polymerization in Randomly Moving Wild-Type and *pikI*^−^ Cells, Related to Figure 1 Random motility of a starved Ax2 and *pikI*^−^ cell expressing Lifeact-GFP, a marker for F-actin. Acquisition frame rate is one frame per second.

Movie S3. Activation of Ras in cAMP-Stimulated Wild-Type and *pikI*^−^ Cells, Related to Figure 3 An Ax2 and a *pikI*^−^ cell expressing Raf1-RBD-GFP (a marker for activated Ras), uniformly stimulated with 1 μM cAMP. Acquisition frame rate is one frame per second; movie begins 5 s before addition of cAMP to the well.
