# Supplementary material for: A PIP5 Kinase Essential for Efficient Chemotactic Signaling
Source: Curr Biol. 2014 Feb 17;24(4):415–21. doi: 10.1016/j.cub.2013.12.052 (PMC3969243; doi:10.1016/j.cub.2013.12.052)
Supplement: Document S1. Figures S1–S3 and Supplemental Experimental Procedures [file mmc1.pdf]

Current Biology, Volume *24*

Supplemental Information

A PIP5 Kinase Essential for Efficient Chemotactic Signaling

Louise Fets, John M.E. Nichols, and Robert R. Kay

Supplemental Information

Supplemental data

Figure S1, relates to Figure 1

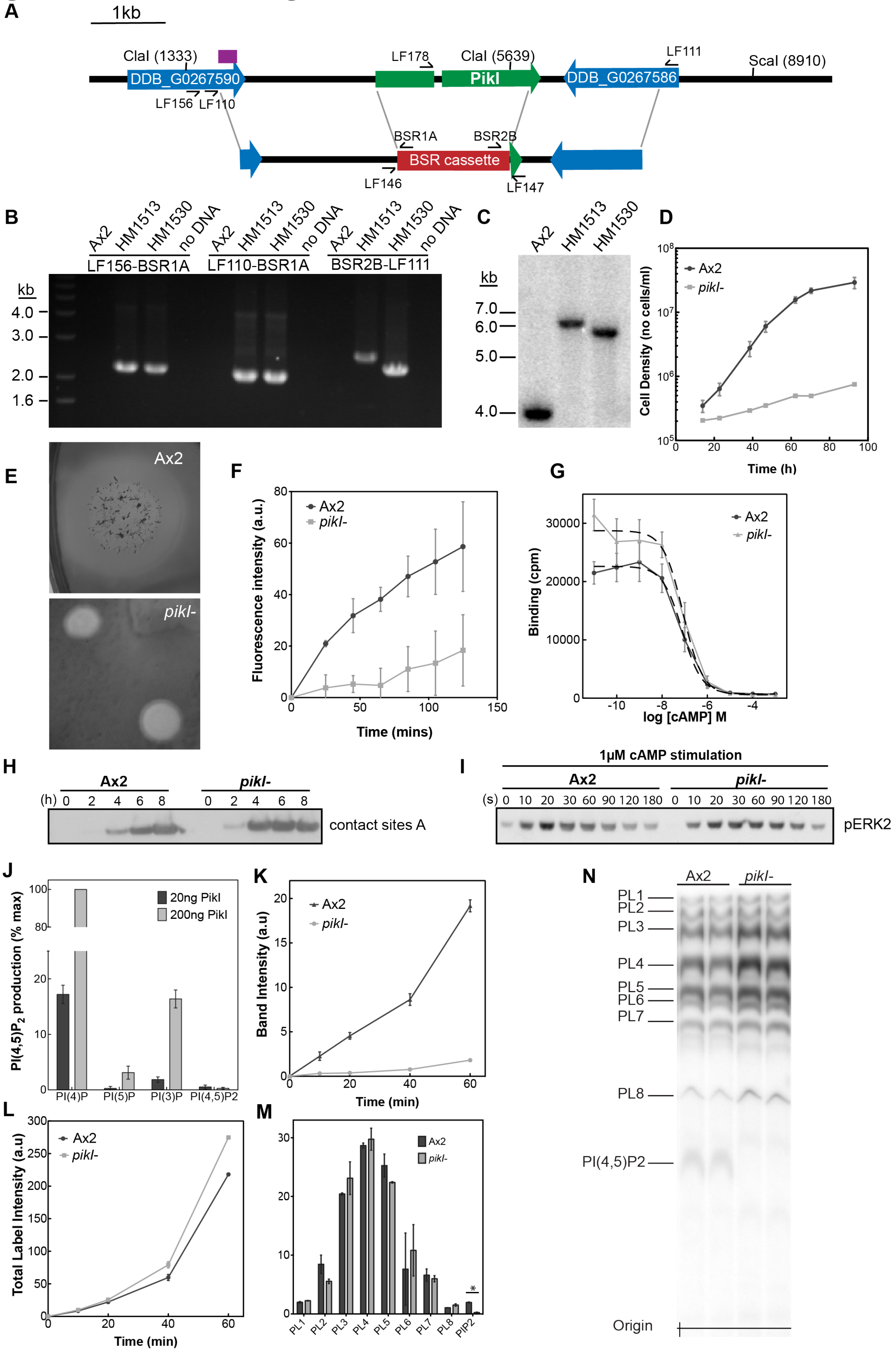

Figure S2, relates to Figure 2

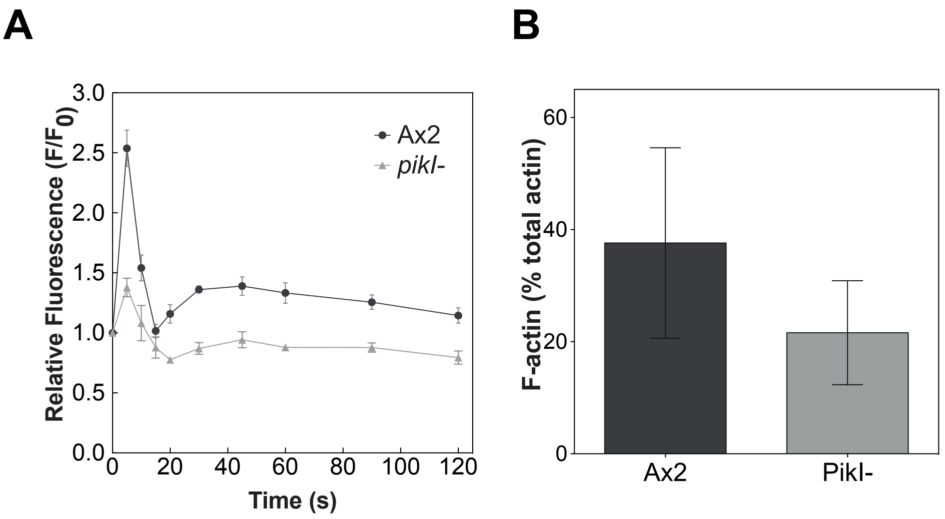

Figure S3, relates to Figure 3

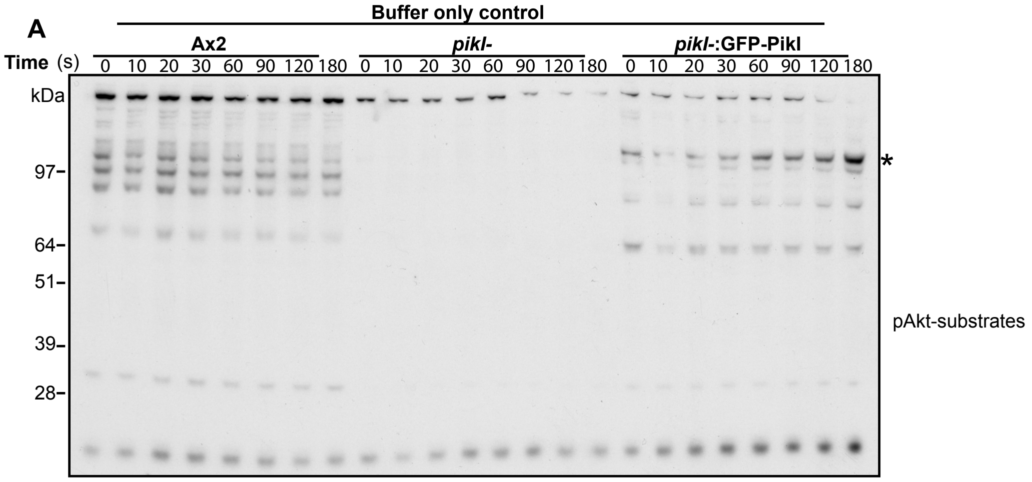

B

	Total Ras (relative)	Supernatant (relative)	Pellet (relative)
**Ax2**	1 (n/a)	0.55 (±0.10)	0.43 (±0.15)
pikl-	1.23 (±0.46)	0.95 (±0.06)	0.39 (±0.15)

Supplemental Figure legends

Figure S1 (related to Figure 1)

A) The *pikI* locus (green) and flanking genes (blue) and disruption cassette. Primers for PCR screening are labelled. Southern blotting strategy is also shown, with restriction sites and probe (purple) indicated. B) PCR products showing two independent knock-out strains; HM1513 and HM1530. Both strains have similar phenotypes, but for clarity, only HM1513 is discussed further here. In all primer sets, a band is only produced when the BSR cassette has integrated into the correct locus, since one primer is within the BSR cassette and the other is beyond the arm of homology. LF156-BSR1A, expected band size 2.3 Kb; LF110-BSR1A, 2.15Kb; BSR2B-LF111, 2.5Kb. The discrepancy in band size between the two clones in this final PCR is due to recombination within the terminator of the blasticidin cassette, which results in loss of 265 bp of terminator sequence. This seems to occur relatively frequently in our hands but does not affect blasticidin resistance. C) Southern blot (after cutting with ClaI and ScaI). Expected wild type band size is 4.3 Kb, while deletion of the ClaI site in the mutant leaves a band at 6.9 Kb. Again, a discrepancy in band size is visible between the two clones. D) Representative growth curve in HL5 media. Data show mean ± SD E) Colony size of Ax2 and *pikI-* clones after 5 days of growth. F) Phagocytosis of TRIT-C labeled yeast, normalized to time zero. Data represent 3 independent experiments (mean±SD) G) Binding of tritiated cAMP to cAMP receptors in the presence of increasing concentrations of unlabelled cAMP. Data obtained in three independent experiments, error bars are ± SD. H) Representative western blot of cell lysates taken at different stages during early development, to show expression of the developmental marker contact sites A

(csaA). I) Representative western blot showing cell lysates from a time-course after cAMP stimulation (at time zero). Blot probed with an antibody that recognises phosphorylated Erk2. J) Quantification of PI(4,5)P_2 production using a range of different substrates and 20ng or 200ng of recombinant PikI, relative to the amount produced by 200 ng PikI using PI(4)P as a substrate. Data are mean ± SEM, from three independent experiments. K) Quantification of relative label uptake into PI(4,5)P2 over 60 minutes (^{32}Pi added at time zero). L) Quantification of relative total label uptake into phospholipids over 60 minutes. Graphs K and L are representative of three independent repeats, error bars are ±SD calculated from duplicates of each time point on the day. M) Quantification of all phospholipids labeled during equilibrium $^{32}P_I$ labeling, each is shown as a percentage of the total ^{32}P label in that TLC lane. Only PI(4,5)P_2 levels were found to be significantly different between Ax2 and *pikI-* cells (data represent means ± SD of two separate experiments using starved cells, with duplicates in each; significance measured using multiple T-tests with Sidak-Bonferroni multiple comparisons correction). N) Example TLC after equilibrium labeling of phospholipids.

Fig S2 (related to Figure 2)

A) Changes in relative F-actin levels in response to cAMP stimulation. Data represent mean ± SEM, n=3 B) Quantification of F-actin levels in resting cells as a percentage of total actin in whole cell lysates (mean ±SD, n=3)

Fig S3 (related to Figure 3)

A) Basal phosphorylation of PKBA/PKBR1 substrates is reduced in *pikI-* cells Representative western blot showing a buffer-control time course, probed with anti-phospho-Akt-substrates, which recognises the phosphorylated substrates of PKBA

and PKBR1. B) Table showing quantification of Ras localisation after cell fractionation and western blotting. Equivalent protein levels were loaded. Numbers represent mean (± SD, n=3).

Supplemental Experimental Procedures

Reagents and Antibodies

Reagents were purchased from Sigma-Aldrich unless otherwise stated. Lipids were from Echelon Biosciences, radiochemicals from Perkin Elmer-NEN. Antibodies for detection of PKBA/PKBR1 substrate phosphorylation - (RXXS*/T*; 110B7E) Rabbit mAb (cat #9614); PKBA and PKBR1 activation loop - Phospho-PKC (pan) (zeta Thr410) (190D10) Rabbit mAb (cat #2060), and PKBR1 hydrophobic motif - Phospho-(Ser/Thr) PDK1-Docking Motif (18A2) Mouse mAb (cat #9634) were from Cell Signalling Technologies. Anti-Pan-Ras (Ab-3) Mouse mAb (RAS 10) was purchased from Calbiochem. Anti-actin was purchased from Sigma (cat #A2066). Anti phosphor-Erk (anti-phospho-p44/p42 MAPK rabbit Ab) was purchased from Cell Signalling Technology (cat #9101). Anti-Contact Sites A was provided by the Developmental Studies Hybridoma Bank. Anti-PKBR1 and anti-Tubulin A [S1] were kind gifts of R.A Firtel, and M. Koonce respectively. RasC and RasG antibodies were kindly provided by G. Weeks.

Dictyostelium gene disruption, DNA constructs and cell culture

All strains were derived from Ax2 (Kay Lab) and grown at 22°C, either in HL5 axenic medium (Formedium) or in association with *Klebsiella aerogenes* on SM agar plates[S2] as appropriate. Note that *pikI-* cells do not grow axenically. When directly comparing Ax2, *pikI-* and *pikI-:GFP-PikI* strains, Ax2 and *pikI-* cells were transferred from *Klebsiella* plates into HL5 (supplemented with heat-killed bacteria) overnight before use. To prepare aggregation-competent cells, vegetative amoebae were harvested and washed in KK2 (16.5 mM KH_2PO_4, 3.9 mM K_2HPO_4, 2 mM $MgSO_4$,

pH 6.1) supplemented with 0.1 mM $CaCl_2$. Cells were resuspended at 2 x 10^7/ml, shaken at 22°C, 180 rpm for one hour and pulsed with 90 nM cAMP every six minutes for four hours.

PikI- cells (strains HM1513 and HM1530) were generated by homologous recombination using vector pLF24 (based on pLPBLP[S3]). Primers LF96 (GCGGGCCCGGTAGATCTCAACAAAGCTCCCTCTTCACTAGAGC) and LF97 (GGAAGCTTGGCGCAAATACAATTGATTCACTAAGTGCTGGG) were used to amplify the 5' arm of homology as an ApaI/HindIII fragment. LF98 (GCAATGCGCGGCCGCCCTTGGTATCATTGATATTTTAATGTTGTATAGTTT ACG) and LF99 (GCTTAGCCGCGGGGGTTGTAATAAAGACGGTAAACTAGG) were used to amplify the 3' arm as a NotI/SacII fragment. The gene was almost entirely deleted, leaving only 160 bp at the 3' end of the gene. Cells were transformed by electroporation[S4] and resistant clones selected by growth in heat-killed bacteria with 15 μg/ml blasticidin. GFP:PikI was expressed in Dictyostelium under the control of the endogenous PikI promoter (pLF46). PikI was cloned from cDNA as an EcoRI/XhoI fragment, and the 1800 bp between PikI and the upstream gene was used as the promoter (cloned as an XbaI/ BglII fragment). Recombinant PikI was expressed using pLF40 (PikI sub-cloned as an EcoR1/XhoI fragment into pGEX6P1(GE healthcare)). Mutated versions were produced by site-directed mutagenesis and cloned into pGEX6P1 to produce pLF41 (pikI- T262A) and pLF42 (pikI- T262E).

Lifeact-GFP was expressed from plasmid pLF63 (BamH1/Xho1 fragment from pDM1005 cloned into BamH1/Xho1 cut from pDM625) this replaced resistance cassette to Actin14 based one, which aids expression in bacteria. pDM1180 contains

the RBD of Raf1 under the control of the actin15 promoter. It contains hygromycin resistance marker under control of the coactosinA promoter. pDM1005, pDM625 and pDM1180 were kind gifts from Dr. Douwe Veltman, MRC-LMB. Cells were transformed by electroporation, and then selected using 60 µg/ml Hygromycin using heat-killed *E. coli* in KK2 as a nutrient source. After 3 days, cells were harvested and used for experiments. If there were insufficient cells to pulse with cAMP, extra, non-transformed cells of that strain were used to make up the correct density, and only fluorescent cells were imaged. By using the cells so quickly after transformation, phenotype reversion was avoided (checked by looking at colony size on *Klebsiella* plates).

Dictyostelium strains used in this study

Strain description	Systematic name	Relevant genotype	Dictybase strain ID
Wild type	Ax2		DBS0235521
pikI-	HM1513	*pikI*	
pi3k1-5-	HM1264	*pikA2, pikB2, pikC2, pikF2, pikG2*	
pi3k1-5-/plc-	HM1475	*pikA2, pikB2, pikC2, pikF2, pikG2,plcA*	

Chemotaxis assays and microscopy

Dunn Chamber: Chemotaxis of aggregation competent cells was imaged using a Dunn Chamber [S5]. 1 µM cAMP was used in the outer chamber. Mutant and wild-type cells were seeded onto either side of a coverslip to allow imaging in the same chamber, under equal gradient conditions. Images were taken every 30 seconds and multiple movies obtained simultaneously using a motorised stage (Prior Scientific) on a Zeiss Axiovert S100 inverted microscope.

Random Motility: Motility of vegetative cells was imaged as above, but in the absence of chemoattractant. Cells were filmed within 30 minutes of washing free from bacteria, and images were taken every 15 seconds.

Statistics were calculated using one-way ANOVA of the mean values obtained for each parameter on each day, so that n= the number of days of imaging, rather than the number of cells tracked.

Micropipette: Chemotaxis towards a micropipette (Eppendorf Femtotips II) filled with 10 µM cAMP (steep gradient) was imaged using an inverted Zeiss 710 confocal microscope with a micromanipulator system (Eppendorf 5171). Images were taken every 15 seconds. After tracking, data from cells initially less that 100 µM from the micropipette were compared with those further than 100 µM from the needle. A 2-way ANOVA (strain, and initial distance from needle) was then used to determine statistical significance, with n representing the number of cells

All movies were analysed using the Manual Tracking and Chemotaxis Tool plug-ins for ImageJ (NIH). Speed is defined as accumulated distance divided by time, persistence as the Euclidean distance divided by accumulated distance and chemotactic index as the cosine of the angle between the net distance travelled in the direction of the gradient and the Euclidean distance.

Imaging F-actin and localization of active Ras: Lifeact-GFP and Raf1-RBD-GFP were imaged in cells either using an inverted Zeiss LSM 710 or Zeiss LSM 780 confocal microscope, and a frame rate of 1frame/second. To image cAMP uniform stimulation, cells were seeded into 8 well Nunc chambered-coverslides, in a volume of 250 µl. After 10-20 s of filming, 50 µl of 6 µM cAMP was carefully added to the well, to generate rapid mixing, and give a final concentration of 1µM. To look at localization of the Raf1 Ras-binding domain, the micropipette was filled with 1 µM cAMP, and images were obtained at a frame rate of 1 per second.

Global Stimulation assays

All assays detailed below were carried out with aggregation-competent cells that had been basalated by shaking with 3 mM caffeine for 30 minutes to prevent endogenous cAMP relay. The cells were washed [KK2 (16.5 mM KH2PO4, 3.9 mM K2HPO4, 2 mM MgSO4, pH 6.1), plus 0.1 mM $CaCl_2$] before use. Unless otherwise stated, cells were stimulated with a final concentration of 1 µM cAMP.

PI(3,4,5)P_3 measurement: After stimulation, cells were quenched with 0.5 M ice cold TCA at the indicated time point. Neutral lipids were extracted first with 2:1 MeOH/$CHCl_3$ and discarded, followed by acidic lipid extraction with MeOH:$CHCl_3$:12M HCl (80:40:1). The acidic lipids were removed to a clean tube, phases split and the organic phase dried down under vacuum. PI(3,4,5)P_3 levels were measured using an ELISA-based mass assay (Echelon Biosciences), following manufacturers instructions.

F-Actin polymerisation assay: F-actin content was determined after stimulation by extracting the triton-insoluble cytoskeleton and staining with TRITC-labelled phalloidin as detailed previously[S6]. Fluorescence was measured using a fluorimeter (Perkin Elmer LS50B).

Calcium Uptake: Calcium uptake after stimulation was measured as described previously[S7].

cAMP accumulation: cAMP accumulation was measured after stimulation with 15 µM 2-deoxy-cAMP. Signalling was quenched at the appropriate time point by addition of perchloric acid (final concentration 1.75 %) before neutralising with 50 % saturated $KHCO_3$. cAMP levels were then measured using a competitive binding assay as detailed previously[S8].

cGMP production: Stimulated cells were quenched at the appropriate time point in the same way as when measuring cAMP accumulation. cGMP levels were measured using a radioimmuno-assay kit (Perkin Elmer), following manufacturers instructions, with ^{125}I detected using a scintillation counter.

Immunoblotting: After stimulating, signalling was quenched at the indicated time point by addition of 3 x LDS sample buffer (Invitrogen) supplemented with 2-mercaptoethanol, protease inhibitors (Roche complete) and phosphatase inhibitors (Sodium pyrophosphate, sodium orthovanadate and ß-glycerophosphate). Standard western blotting procedures were then used. All membranes were blocked with 5 % BSA and antibodies were used following manufacturers instructions.

Ras activity assay: Ras activity was measured as detailed previously [S9]. Cells were pulsed for 5 hours rather than 4 (measurements were found to be more robust at this point in development). Briefly, cells were stimulated and then lysed at the appropriate timepoint with 2 x HKLB (20 mM Sodium phosphate buffer pH 7.2, 300 mM NaCl, 20 mM $MgCl_2$, 2 mM EDTA, 2 mM sodium orthovanadate, 10 mM sodium fluoride, 2 % Triton-X-100, 20 % glycerol and protease inhibitors (Roche complete, 2 per 50 ml)). 400 µg protein was incubated with 50 µl (50 % slurry) of GST-Byr2-RBD bound glutathione sepharose 4B beads for 1 hour at 4°C. Beads were washed with 1 x HKLB, resuspended in LDS sample buffer and boiled for 5 minutes. Samples were blotted with anti-pan-Ras antibody (see above) and bands were quantified using a Biorad Chemidoc XRS+ system and associated ImageLab software.

Resting F-actin Measurement

Aggregation-competent cells were washed twice in KK2 and basalated in 5 mM caffeine for 30 minutes before being washed twice with KK2 and resuspended at $1x10^8$ cells per ml. Triton-insoluble cytoskeletons were prepared as detailed previously [S10].

Cytoskeleton pellets were resuspended in LDS sample buffer (plus protease inhibitors). Whole cell lysates (from equivalent cell numbers) were prepared from cells pelleted by brief centrifugation and resuspended in LDS sample buffer (plus protease inhibitors).

Dilutions of cytoskeleton samples (0.5, 0.33, 0.25) and whole cell lysates (0.1, 0.2, 0.3, 0.4, 0.5, 0.6, 0.8, 1.0) were separated by SDS-PAGE and western blotting with anti-actin was used to quantify relative F actin (in cytoskeleton pellets) compared to total actin (in whole cells lysates). Quantification of western blots was done using a Biorad Chemidoc XRS+ system and associated ImageLab software

Protein purification

Constructs pLF40, 41 and 42 were expressed in BL21-CodonPlus (DE3)-RIL *E. coli*, and induced overnight at 18°C using 200 µM IPTG. Cells were lysed in buffer (50 mM Tris-HCl [pH 7.4], 100 mM NaCl, 0.2 % NP40, 1 mM EDTA, 2 mM DTT, 10 % Glycerol, protease inhibitor tablets [Roche complete EDTA free - 1 in 50ml]) using an Emulsiflex C5 (Avestin), and protein was purified using glutathione sepharose 4B beads (GE Healthcare). GST tags were cleaved from the proteins on the column using Prescission protease (GE Healthcare).

PIP-Kinase assays

PIP-kinase assays were carried out as described[S11], except that enzymatic reactions took place at 22°C to reflect the growth temperature of *Dictyostelium*. TLCs were imaged using a Typhoon phosphorimager (GE Healthcare) and bands quantified using ImageQuant (Molecular Dynamics). Michaelis-Menten curves were fitted in Graphpad Prism.

Competitive [^{3}H]-cAMP binding assay

cAMP receptor numbers were determined using an assay described previously[S12]. Briefly, aggregation-competent cells (resuspended at 2 x 10^8/ml) were mixed with an equal volume of cAMP assay buffer (50 nM [2-,8-^{3}H]-cAMP in KK2) containing unlabelled cAMP ranging from 20 pM to 2 mM. Samples were incubated on ice for 30 s. 1.2 ml saturated $(NH_4)_2SO_4$ was added to stabilise binding. Cells were pelleted, resuspended in 1% SDS and samples counted on a scintillation counter. A competitive-binding curve (fitted using GraphPad Prism) was used to calculate K_D and receptor numbers.

Phagocytosis assay

This method has been described before[S13]. Briefly, cells were harvested from clearing plates and washed 4-5 times in KK2 to remove bacteria. They were resuspended at 2 x 10^6 per ml in axenic medium. 20 ml of cells were shaken in a conical flask at 180 rpm for 15 minutes before adding TRITC-labeled yeast. Samples were taken immediately and added to filtered trypan blue solution (2 mg/ml trypan blue in 20 mM citrate pH 4.5, 150 mM NaCl). They were then agitated for 3 minutes

before pelleting cells. Supernatant was discarded and cells were resuspended in 1 ml KK2. Fluorescence was measured using a fluorimeter (544 nm excitation and 574 nm emission). Further samples were taken at 25, 45, 65, 85 and 105 minutes. Increase in fluorescence was plotted against time after subtracting the background fluorescence (value obtained at t=0).

Radio-labelling of phospholipids

For short-term labelling, aggregation-competent cells were shaken at $2x10^7$/ml in phosphate-free buffer (10 mM MES pH 6.2, 10 mM KCl, 10 mM NaCl, 1 mM $CaCl_2$, 100 μg/ml dihydrostreptomycin) and labelled with 20 μCi/ml ^{32}P-inorganic phosphate. At various times 400 μl samples were taken, quenched with an equal volume of ice-cold 1 M trichloroacetic acid, incubated on ice for 10 min, centrifuged briefly and the supernatant discarded. Pellets were extracted with 700 μl 80: 40: 1 methanol/chloroform/HCl by vortexing and bath sonication, and the phases split with 420 μl 0.1 M HCl and 233 μl chloroform. The organic phase was dried under vacuum, taken up in 2:1 methanol/chloroform, analysed by TLC using Whatman LK6D TLC plates pre-dipped in 1 % potassium oxalate (in 50 % methanol) and baked at 110°C for at least one hour. Running solvent was 28:40:6:10 chloroform/methanol/ammonia/water. Plates were analysed using a Typhoon Phosphorimager and ImageQuant software (GE Healthcare). PI(4,5)P_2 was identified using authentic standards purified from pig brain (kind gift of Robin Irvine) or radioactively synthesised using purified PikI and di-C_{16}-PI4P or extracted *Dictyostelium* lipids. Equilibrium labelling was carried out as described for short-term labelling, except 10 μCi/ml ^{32}P-inorganic phosphate was added to cells during growth in suspension with heat-killed bacteria (in labelling buffer) and cells were

labelled for more than four generations before their lipids were extracted and analysed.

Cell Fractionation

Aggregation-competent cells were washed and resuspended in ice-cold lysis buffer (20 mM KCl, 50 mM HEPES (pH7.0), 2 mM $MgSO_4$, 1 mM EDTA, 1 mM DTT, 10% glycerol, 1 tablet of Roche complete protease inhibitors per 50ml buffer) and then filter lysed by passing first through a pre-filter and subsequently a nucleopore filter (3 μM pore size). Lysate was centrifuged briefly to remove whole cells and then centrifuged for 30 minutes at 75,000 x g, 4°C. Cell equivalents of whole cell lysate, supernatant and pellet, which corresponded to membrane and cytosol fractions (verified by blotting for membrane protein contact-sites A and tubulin, data not shown) were run on a gel and then western blotted using an anti-pan-Ras antibody. Quantification of western blots was done using a Biorad Chemidoc XRS+ system and associated ImageLab software.

Supplemental References

S1. Piperno, G., and Fuller, M.T. (1985). Monoclonal antibodies specific for an acetylated form of alpha-tubulin recognize the antigen in cilia and flagella from a variety of organisms. J. Cell. Biol. *101*, 2085-2094.

S2. Kay, R.R. (1987). Cell differentiation in monolayers and the investigation of slime mold morphogens. Methods Cell Biol. *28*, 433-448.

S3. Faix, J., Kreppel, L., Shaulsky, G., Schleicher, M., and Kimmel, A.R. (2004). A rapid and efficient method to generate multiple gene disruptions in *Dictyostelium discoideum* using a single selectable marker and the Cre-loxP system. Nucleic acids Res *32*, e143.

S4. Knecht, D., and Pang, K.M. (1995). Electroporation of *Dictyostelium discoideum*. Methods Mol. Biol. *47*, 321-330.

S5. Zicha, D., Dunn, G.A., and Brown, A.F. (1991). A new direct-viewing chemotaxis chamber. J. Cell Sci. *99 (Pt 4)*, 769-775.

S6. Hall, A.L., Schlein, A., and Condeelis, J. (1988). Relationship of pseudopod extension to chemotactic hormone-induced actin polymerization in amoeboid cells. J. Cell Biochem. *37*, 285-299.

S7. Traynor, D., Milne, J.L., Insall, R.H., and Kay, R.R. (2000). Ca(2+) signalling is not required for chemotaxis in *Dictyostelium*. Embo J. *19*, 4846-4854.

S8. Van Haastert, P.J. (2006). Analysis of signal transduction: formation of cAMP, cGMP, and Ins(1,4,5)P3 in vivo and in vitro. Methods Mol. Biol. *346*, 369-392.

S9. Bolourani, P., Spiegelman, G.B., and Weeks, G. (2008). Rap1 activation in response to cAMP occurs downstream of ras activation during *Dictyostelium* aggregation. J. Biol. Chem. *283*, 10232-10240.

S10. McRobbie, S.J., and Newell, P.C. (1983). Changes in actin associated with the cytoskeleton following chemotactic stimulation of *Dictyostelium discoideum*. Biochem. Biophys. Res. Comm. *115*, 351-359.

S11. Hinchliffe, K.A., Giudici, M.L., Letcher, A.J., and Irvine, R.F. (2002). Type IIalpha phosphatidylinositol phosphate kinase associates with the plasma membrane via interaction with type I isoforms. Biochem. J *363*, 563-570.

S12. Liao, X.H., and Kimmel, A.R. (2009). Biochemical responses to chemoattractants in *Dictyostelium*: ligand-receptor interactions and downstream kinase activation. Methods Mol. Biol. *571*, 271-281.

S13. Rivero, F., and Maniak, M. (2006). Quantitative and microscopic methods for studying the endocytic pathway. Methods Mol. Biol. *346*, 423-438.
