# Supplementary figures and images for: A PIP5 Kinase Essential for Efficient Chemotactic Signaling
Source: Curr Biol. 2014 Feb 17;24(4):415–21. doi: 10.1016/j.cub.2013.12.052 (PMC3969243; doi:10.1016/j.cub.2013.12.052)

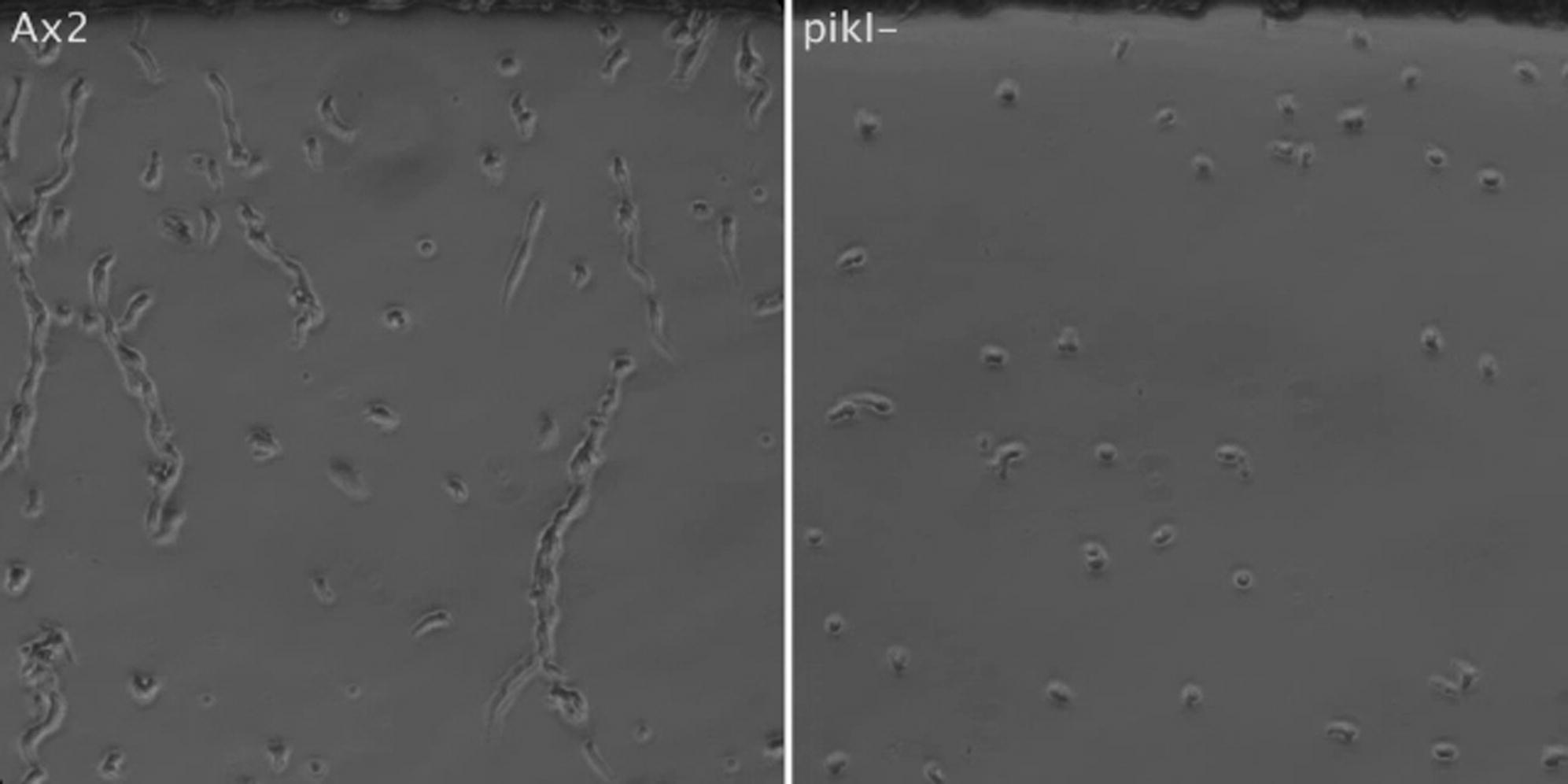

Supplement: Movie S1. Chemotaxis in Wild-Type and pikI− Cells, Related to Figure 1 — Ax2 and pikI− cells chemotaxing toward cAMP in a Dunn chamber (the outer well, filled with 1 μm cAMP, can just be seen at the top of the screen). Acquisition frame rate is two frames per minute. [file mmc2.jpg]

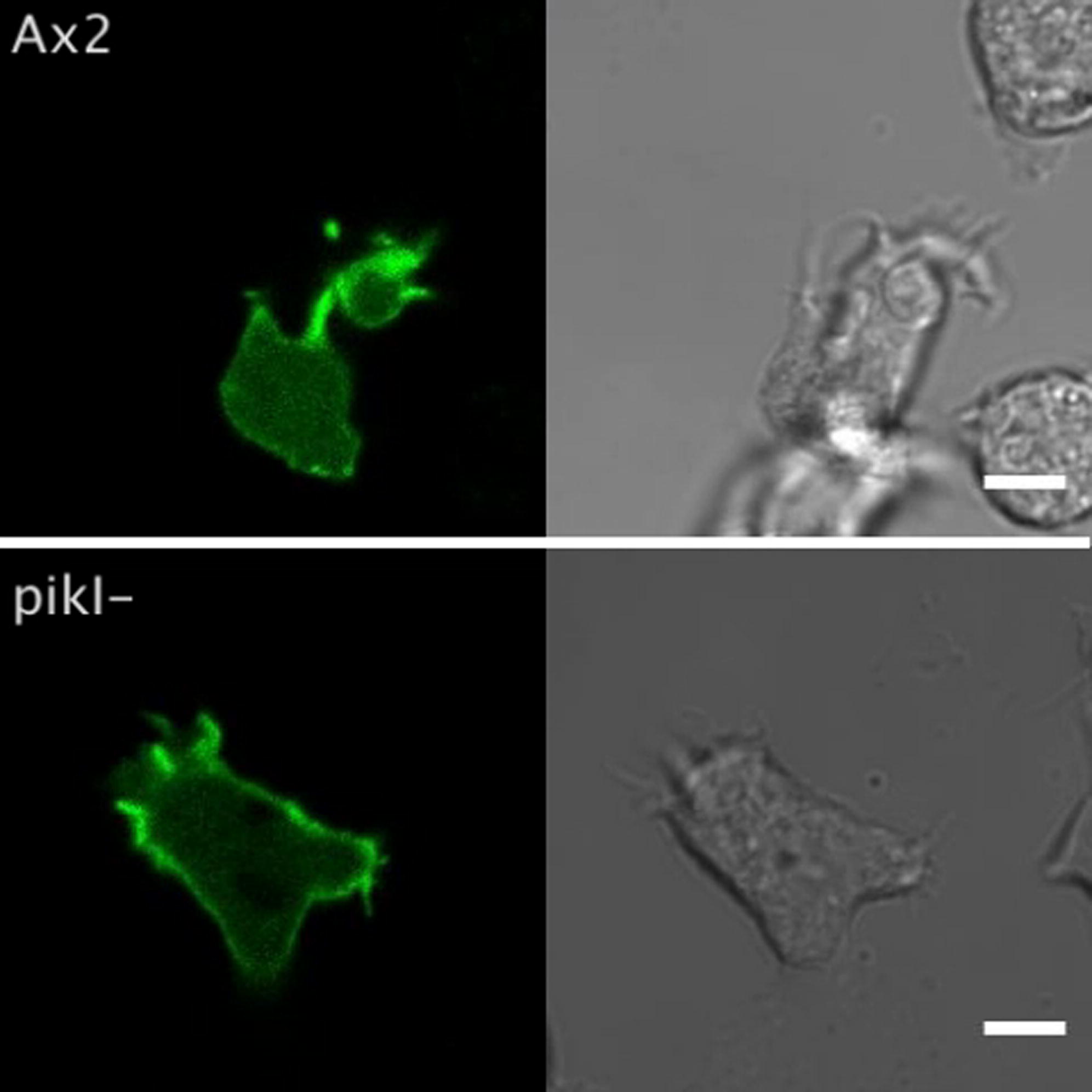

Supplement: Movie S2. Actin Polymerization in Randomly Moving Wild-Type and pikI− Cells, Related to Figure 1 — Random motility of a starved Ax2 and pikI− cell expressing Lifeact-GFP, a marker for F-actin. Acquisition frame rate is one frame per second. [file mmc3.jpg]

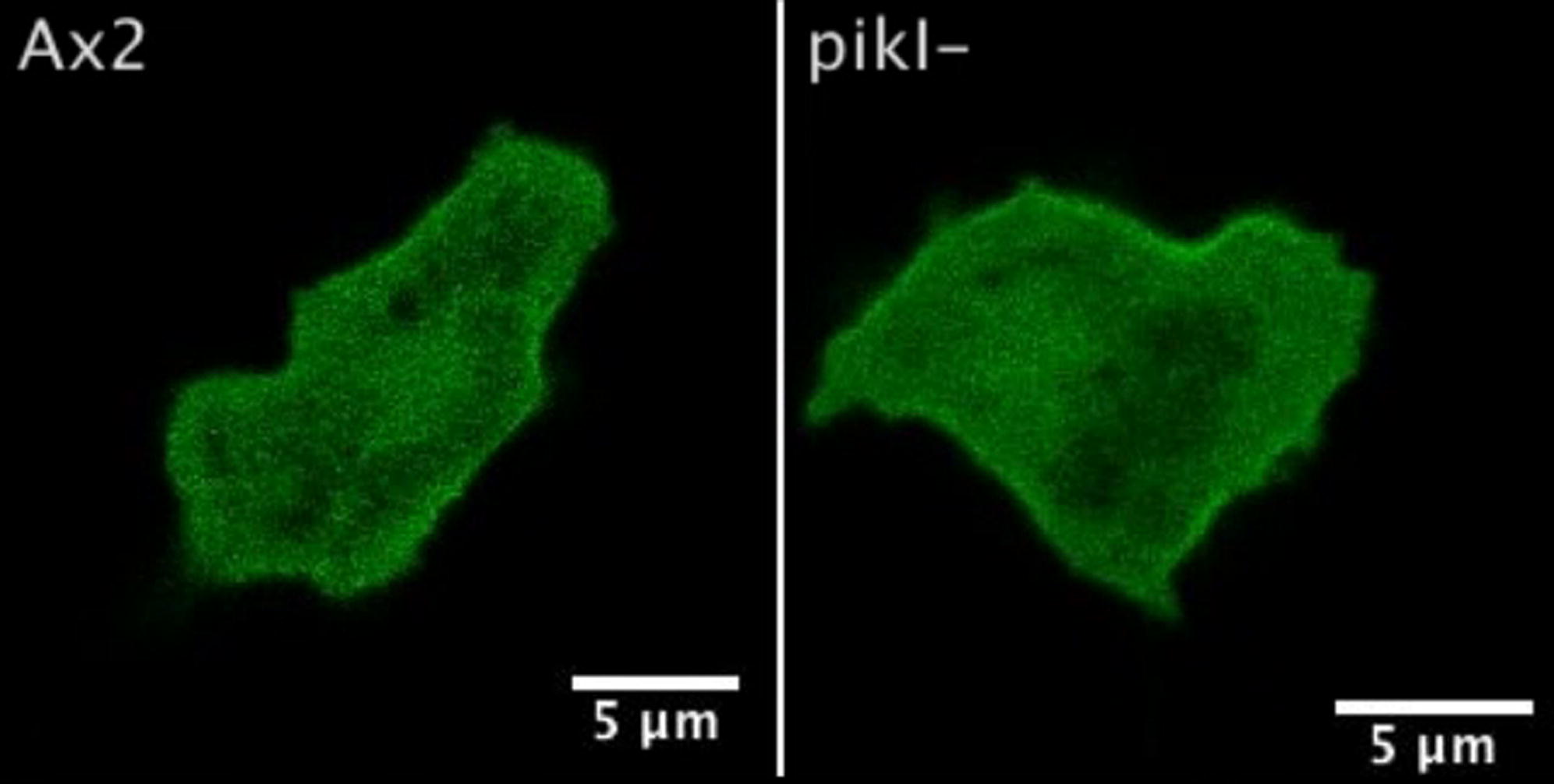

Supplement: Movie S3. Activation of Ras in cAMP-Stimulated Wild-Type and pikI− Cells, Related to Figure 3 — An Ax2 and a pikI− cell expressing Raf1-RBD-GFP (a marker for activated Ras), uniformly stimulated with 1 μM cAMP. Acquisition frame rate is one frame per second; movie begins 5 s before addition of cAMP to the well. [file mmc4.jpg]
